# Identification of Antioxidant Capacity -Related QTLs in *Brassica oleracea*

**DOI:** 10.1371/journal.pone.0107290

**Published:** 2014-09-08

**Authors:** Tamara Sotelo, María Elena Cartea, Pablo Velasco, Pilar Soengas

**Affiliations:** Group of Genetics, Breeding and Biochemistry of Brassicas, Department of Plant Genetics, Misión Biológica de Galicia, Spanish Council for Scientific Research (MBG-CSIC), Pontevedra, Spain; University of Malaga-Consejo Superior de Investigaciones Científicas, Spain

## Abstract

*Brassica* vegetables possess high levels of antioxidant metabolites associated with beneficial health effects including vitamins, carotenoids, anthocyanins, soluble sugars and phenolics. Until now, no reports have been documented on the genetic basis of the antioxidant activity (AA) in *Brassica*s and the content of metabolites with AA like phenolics, anthocyanins and carotenoids. For this reason, this study aimed to: (1) study the relationship among different electron transfer (ET) methods for measuring AA, (2) study the relationship between these methods and phenolic, carotenoid and anthocyanin content, and (3) find QTLs of AA measured with ET assays and for phenolic, carotenoid and anthocyanin contents in leaves and flower buds in a DH population of *B. oleracea* as an early step in order to identify genes related to these traits. Low correlation coefficients among different methods for measuring AA suggest that it is necessary to employ more than one method at the same time. A total of 19 QTLs were detected for all traits. For AA methods, seven QTLs were found in leaves and six QTLs were found in flower buds. Meanwhile, for the content of metabolites with AA, two QTLs were found in leaves and four QTLs were found in flower buds. AA of the mapping population is related to phenolic compounds but also to carotenoid content. Three genomic regions determined variation for more than one ET method measuring AA. After the syntenic analysis with *A. thaliana*, several candidate genes related to phenylpropanoid biosynthesis are proposed for the QTLs found.

## Introduction

*Brassicaceae* plants represent one of the major vegetable crops grown worldwide, with *Brassica oleracea* L. (2n = 18) as the main *Brassica* species consumed in Europe and the USA. Cruciferous vegetables, in particular those included in the *Brassica* genus, are an important part of the diet as they provide a multitude of nutrients and bioactive compounds [1]. A high consumption of *Brassica* vegetables reduces the risk of age-related chronic illnesses, degenerative diseases [2] and several types of cancer [3]. Human health benefits associated to *Brassica* consumption could be attributed, in part, to the large amount of constituents having strong antioxidant activity (AA). In fact, AA of *Brassica* vegetable extracts is higher compared to that of other vegetable crops like green pepper, carrot, potato or green bean [4]. Antioxidants have long been recognized to have protective functions against oxidative damage and are associated with a reduced risk of chronic diseases [5]. *Brassica* vegetables possess high levels of antioxidant metabolites associated with beneficial health effects, including vitamins (especially vitamin A, C, E, K and B-6), carotenoids (such as γ- and β-carotene and zeaxanthin), anthocyanins, folate, soluble sugars and phenolic compounds which are known to be the major antioxidants of *Brassica* crops [6]–[14].

Due to the complexity of food composition, separating each antioxidant compound and studying it individually is costly and inefficient. In addition, there might be synergistic interactions among the antioxidant compounds [15]. There are numerous methods for measuring the total AA of a plant extract *in vitro*. The 2- single electron transfer reaction based assays (ET) measure the reducing capacity of the samples. The ET group includes different methods like the ferric ion reducing antioxidant power assay (FRAP), and the AA measured with the reagents ABTS (2, 2′-azino-bis (3-ethylbenzthiazoline-6-sulphonic acid)) and DPPH (2, 2-diphenyl-1-picrylhydrazyl), among others [15]. Generally speaking, correlations found among these three methods are high in Brassica extracts. Soengas *et al.* [16] found that the correlation between DPPH and FRAP was 0.8 when analyzing several *B. oleracea* crops. Kusznierewicz *et al.* [17] found a correlation of 0.96 between ABTS and DPPH in white cabbage. Zhi *et al.* (2011) [18] found correlations ranging from 0.76 to 0.82 among the three cited methods when analyzing different vegetables, including broccoli. In most studies, several ET methods are often used in order to measure the AA of a sample, but theoretically it could be possible to choose only one because of the high correlations among assays.

Phenolic compounds are known to be the major group with antioxidant capacity in *Brassica* crops [13]. These compounds are able to scavenge reactive oxygen species due to their electron donating properties. The most widespread and diverse group of polyphenols in *Brassica* species are flavonoids and hydroxycinnamic acids. In many *in vitro* studies, phenolic compounds demonstrated higher AA than other antioxidants, such as vitamins and carotenoids [19].

Several studies have demonstrated that highly pigmented cultivars of some vegetables (i.e. cabbage, cauliflower) possess stronger AA than their respective light-colored cultivars [20]–[22]. This could indicate that pigments ‘*per se*’ have AA. Carotenoids are a diverse group of more than 600 natural pigments that accumulate in the plastids of some vegetables leaves, flowers and fruits [23]. Some carotenoids are essential nutrients for humans, while others have protective effects against several diseases. Anthocyanins are natural pigments responsible for the blue, purple, red and orange colors in the major parts of all higher plants and have attracted much interest due to their impact on the sensorial characteristics of food products, as well as their health-related properties through various biological activities [24], [25]. The AA of *Brassica* crops has been mainly related to phenolic compounds and vitamin C. However, carotenoids and anthocyanins could also play an important role.

Comparisons of *in vitro* AA of the main *B. oleracea* crops demonstrated that broccoli, kale and red cabbage show high AA [17], [26]. Soengas *et al.* [16] compared the AA of six *Brassica* crops, including broccoli, cabbage, cauliflower, kale, nabicol and tronchuda cabbage, at four different plant stages with DPPH and FRAP assays. They found that kale and broccoli had the highest AA. Nilson *et al.* [27] found that AA of curly kale was at least 10-fold higher than that of cauliflower and white cabbage. At present, there are many studies about AA of *Brassica* crops because of the health related properties of antioxidants. However, as far as we know, there are no repots about genetics and heredity associated with AA in the *Brassica* genus.

QTL analysis is a very important tool in order to study the genetic base of AA. For the last decades, quantitative trait mapping has been the most common approach in order to analyze complex traits and measure the association of genetic markers with phenotypic variation. Identification of QTLs is essential for the understanding of the quantitative genetic control of AA and it is an early step in order to identify and estimate the gene number controlling each trait variation. The high co-linearity between *A. thaliana* and *Brassica* species can be used for identifying candidate genes underlying QTLs that affect AA. To our knowledge, this is the first report on the genetic basis of AA in *Brassica* crops. In other crops, only Jin *et al.* [28] in rice, Dobson *et al.* [29] in raspberry and Hayashi *et al.* [30] in lettuce studied QTLs for total water soluble AA and total phenolic, anthocyanin and carotenoid contents.

For this reason, the aims of our research were 1) to study the relationship among different ET methods for measuring AA, 2) to study the relationship between these methods and phenolic, carotenoid and anthocyanin contents and 3) to find QTLs of AA measured with ET assays and for phenolic, carotenoid and anthocyanin contents in two organs of a DH population of *B. oleracea* as an early step in order to identify genes related to these traits.

## Materials and Methods

### Chemicals

DPPH (2,20-diphenyl-1-picrylhydrazyl), TPTZ (2,4,6-tripyridyl-striazine), Trolox (6-hydroxy-2,5,7,8-tetramethylchroman-2-carboxylic acid), hydrochloric acid, phenolics reagent, ABTS (2, 2′-azino-bis (3-ethylbenzthiazoline-6-sulphonic acid)), potassium persulphate and gallic acid were obtained from Sigma–Aldrich Chemie GmbH (Steinheim, Germany); ferric chloride and methanol were obtained from Panreacquimica S.A. (Castellar del Vallés, Spain).

### Plant material and growing environments

The double haploid (DH) mapping population employed in this study (BolTBDH) was created from an F_1_ individual, derived by crossing a DH broccoli line ‘Early Big’(P_2_) and a DH rapid cycling of Chinese kale line (TO1000DH3,P_1_) [31]. Parents and 155 DH lines were grown in autumn 2011 (from September to November) and stored in the greenhouse under controlled conditions: 16 h of daylight and a temperature of 24±2°C; 8 h of darkness having 18±2°C at night; and a relative humidity of 55% in order to obtain enough seed in the same environmental conditions. Plants were sown in a completely randomized experiment with two replications and four plants per replication. Two sample types were collected and analysed: leaves (one month after sowing) and flower buds (taken sequentially depending on the maturity of each line). Bulks of individual samples were taken from each replication. Samples were frozen *in situ* in liquid N_2_, immediately transferred to the laboratory and frozen at −80°C. All samples were freeze-dried (BETA 2–8 LD plus, Christ) for 72 h. The dried material was powdered by using an IKA-A10 (IKA-Werke GmbH & Co.KG) mill, and the fine powder was used for methanolic extractions.

### Evaluation of AA

Freeze-dried and ground samples (10 mg) were extracted with 1 ml of 80% aqueous methanol in dark maceration for 24 h. After centrifugation (3700 rpm, 5 min), methanolic extracts were employed in order to determine AA (FRAP, DPPH and ABTS) of the mapping population. All AA assays and the content of metabolites with AA were carried out spectrophotometrically by using a microplate spectrophotometer (Spectra MR; Dynex Technologies, Chantilly, VA). Two repetitions were made for each sample and analysis. Standards prepared with different concentrations of Trolox (0, 0.008, 0.016, 0.024, 0.032, 0.04 mM) were measured for FRAP, DPPH and ABTS analyses and AA values were normalized to Trolox equivalents per gram of dry weight.

### FRAP assay

The ferric reducing antioxidant activity (FRAP) assay of Benzie and Strain [32] was measured in all samples. Fresh FRAP reagent was prepared by mixing 10 volumes of 300 mM acetate buffer (pH 3.6), one volume of 10 mM TPTZ in 40 mM hydrochloric acid and one volume of 20 mM ferric chloride, and then incubating at 37°C for 5 minutes. For each analysis, 30 µl of methanolic solution of the two organs (leaves and flower buds) were added to 20 µl of distilled water and 250 µl of fresh FRAP solution and mixed thoroughly. The increase in absorbance was recorded at 593 nm after 20 min.

### DPPH radical scavenging activity

The antioxidant activity by the DPPH method was determined by monitoring the disappearance of the radical DPPH spectrophotometrically, according to Brand-Williams *et al.* [33]. The working DPPH reagent was prepared by dissolving DPPH in methanol to a final concentration of 75 µM. Fifty microliters of extract for leaves and 35 µl for flower buds were added to 250 µl of freshly prepared DPPH reagent and mixed thoroughly. Readings were taken at 517 nm after 30 min of incubation in the dark at room temperature.

### ABTS+ radical scavenging activity

The method of decolorization of free radical ABTS+ employed was a modified version of that used by Samarth *et al.* [34] and initially reported by Re *et al.* [35]. ABTS+ was generated by oxidation of ABTS 7 mM with potassium persulphate 2.45 mM in water, at room temperature for 16 h. For each analysis, the ABTS+ solution was freshly diluted with water in order to obtain an initial absorbance around 0.8 at 734 nm. An aliquot of 20 µl methanolic extract for leaves and 30 µl for flower buds were added to 250 µl of ABTS+ solution. Absorbances were measured at 734 nm after 30 min of incubation in the dark at room temperature.

### Quantification of phenolic content

The total phenolic content of the extracts was determined according to the phenolic colorimetric method described by Dewanto *et al.* [36]. The same methanolic extracts employed for AA assays were employed in order to determine phenolic content. Extracts were oxidized with 50 µl of 0.5 M Folin reagent. After 5 min, 200 µl of a 20% Na_2_CO_3_ solution were added in order to neutralize the reaction. Absorbances were measured at 760 nm after 2 h of incubation in the dark at room temperature. Standards prepared with different concentrations of gallic acid (0, 0.008, 0.016, 0.024, 0.032 and 0.04 mM) were also measured. Results were expressed in terms of micromoles of gallic acid equivalents per gram of dry weight.

### Quantification of carotenoid content

Carotenoid content was determined according to Sims & Gamon [37] with minor modifications. Lyophilized samples (10 mg) were ground in 1 ml cold acetone/Tris buffer solution (80∶20 vol:vol, pH  = 7.8). Samples were mixed overnight in the dark at room temperature; afterwards, the absorbance of samples was measured at 537, 647 and 663 nm. Carotenoid content was computed by following the equations of Sims & Gamon [37] and results were expressed in micromoles per gram of dried weight.

### Quantification of anthocyanin content

Anthocyanin content was determined according to Murray *et al.* [38] with minor modifications. Lyophilized samples (10 mg) were ground in 1 ml of cold methanol/HCL/water (90∶1∶1, vol:vol:vol). Samples were mixed overnight in the dark at room temperature. The absorbance of samples was measured at 529 and 650 nm and anthocyanin content was determined by using the equation described in Sims & Gamon [37]. Results were expressed in micromoles per gram of dried weight.

### Statistical and QTL analysis

A combined analysis of variance across organs and individual analyses of variance for each organ were made for the AA content measured ABTS, DPPH, FRAP assays and for phenolic, carotenoid and anthocyanin contents by using the procedure ANOVA of SAS v 9.2 [39]. Parental differences were analyzed one-tail “t” test by using PROC TTEST of SAS v 9.2 [39]. Simple correlation coefficients were computed with PROC CORR of SAS v 9.2 [39] for each trait.

The genetic map created by Iñiguez-Luy *et al.* [31] has 279 markers (SSRs and RFLPs) distributed along nine linkage groups (C1–C9) with a total distance of 891.4 cM and a marker density of 3.2 cM/marker. Quantitative trait locus mapping was carried out through a composite interval mapping method [40] by using PLABQTL [41]. Individual analyses were carried out for each trait and organ (leaves and flower buds). A likelihood odds (LOD) threshold was chosen for each trait in order to declare the putative QTL significant by following a permutation test, with N = 1000, and a critical alpha value of 25%. The confidence intervals were set to 95%. The analysis and cofactor election were carried out by following PLABQTL's recommendations, using an ‘F-to-enter’ and an ‘F-to-delete’ value of 7.

The proportion of phenotypic variance explained for a specific trait was determined by the adjusted coefficient of determination of regression (R^2^) fitting a model which includes all detected QTLs [42]. Fivefold cross-validation of QTLs was performed by following the procedures described by Utz *et al.* [43]. The whole data set was randomly split into k = 5 data subsets. Four of these subsets were combined to form the estimation set (ES). The remaining subset formed the test set (TS), in which predictions derived from ES were tested for their validity by correlating predicted and observed data. We used 1,000 replicate CV/G runs. Estimates of medians and percentiles and the frequency of QTL detection in ES and TS were calculated over all replicated CV/G runs. The frequency of QTL detection gives us an estimation of the precision of QTL localization. The PLABQTL [41] software package was used for all calculations. Iñiguez-Luy *et al.* (2009) identified collinear genomic blocks between the BolTBDH mapping population and *A. thaliana* by using a synteny analysis. This information was employed in order to locate candidate genes which may directly account for QTLs in *B. oleracea.* By following this approach, we searched in the database TAIR (the *Arabidopsis* information resource http://www.arabidopsis.org) genes related to phenylpropanoid biosynthetic process metabolism (phenolic compounds and anthocyanins are synthetized following this pathway) and genes involved in the carotenoid biosynthetic process by including the words ‘phenylpropanoid’ and ‘carotenoid’ into the field 'description of the gene in TAIR. Twenty one genes related to phenylpropanoids and 24 genes related to carotenoids were found. We tried to locate these genes on the BolTBDH map by means of *in silico* mapping.

## Results

### Quantitative variation for methods measuring AA and the content of metabolites with AA

**Figure 1:**
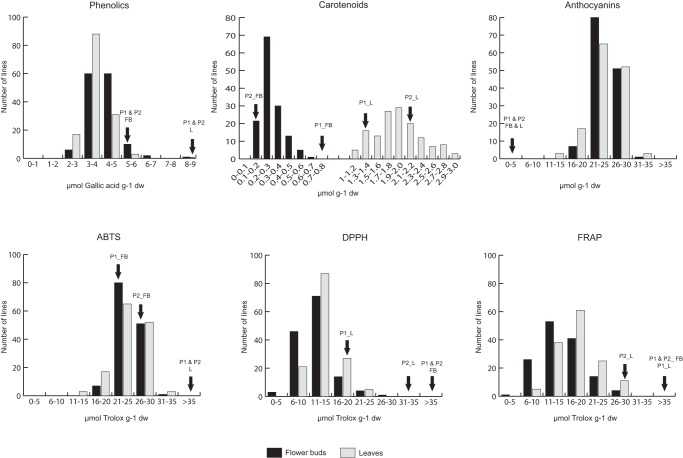
Distribution of the three metabolites with antioxidant activity, carotenoids, anthocyanins and phenolics and the three antioxidant assay methods, ABTS, DPPH and FRAP in the BoITBDH population. Arrows indicate values for the P1 (DH rapid cycling of Chinese kale TO1000DH3) and P2 (DH broccoli line ‘Early Big’) in the two organs under study, leaves (L) and flower buds (FB).

In this study AA in leaves and flower buds was determined by three ET methods: FRAP, DPPH and ABTS. The content of metabolites with AA (phenolics, anthocyanins and carotenoids) was also determined. We used two ET methods (DPPH and ABTS) where the scavenging was followed by monitoring the decrease in absorbance over time, which occurred due to the AA of the sample [44]. For the FRAP assay, the extract shows an increase of absorbance over time dependent on their AA [45]. A transgressive distribution was found for all traits in both organs (Fig. 1). Results obtained from each analysis are considered below.

### FRAP, DPPH and ABTS assays

Mean values for the FRAP and DPPH methods in the population were lower than the corresponding values of ABTS assay in both organs (leaves and flower buds). In leaves, we found mean values of 18.36, 14.04 and 24.78 µmol Trolox g−1 DW in FRAP, DPPH and ABTS assays, respectively. In flower buds, we found values of 15.37, 12.51 and 25.16 µmol Trolox g−1 DW in FRAP, DPPH and ABTS assays, respectively (Table 1).

**Table 1:** Antioxidant activity of parents and population measured in leaves and flower buds with three different antioxidant assay systems and the content of three metabolites with antioxidant activity.

	Leaves			Flower buds		
Traits	P1	P2	Population mean	P1	P2	Population mean
**ABTS** (µmol Trolox g^−1^ DW)	42.06	44.89	24.78	21.13	30.94	25.16
**DPPH** (µmol Trolox g^−1^ DW)	20.20	34.18	14.04	50.65	47.84	12.51
**FRAP** (µmol Trolox g^−1^ DW)	48.17	56.27	18.36	59.40	28.71	15.37
**PHENOLICS** (µmol Gallic Acid g^−1^ DW)	8.02	8.91	3.64	5.55	5.54	4.14
**ANTHOCYANINS** (µmol g^−1^ DW)	0.03	0.67	58.53	0.04	0.13	13.31
**CAROTENOIDS** (µmol g^−1^ DW)	1.48	2.17	1.98	0.84	0.17	0.28

Population mean values between the two organs present highly significant differences for FRAP (F = 75.95, P = 0.0129) and DPPH (F = 65.09, P = 0.0150) methods.

### Metabolites with AA: phenolic, anthocyanin and carotenoid content

Concerning the content of metabolites with AA, we found two different profiles. For the phenolics assay, population showed higher mean values in flower buds than in leaves (4.14 and 3.64 µmol gallic acid g−1 DW, respectively), although differences were not significant. However, both parental lines had higher phenolic content in leaves than in flower buds (Fig. 1).

Leaves of the mapping population had higher anthocyanin and carotenoid content (58.53 µmol g−1 DW and 1.98 µmol g−1 DW, respectively) compared to flower buds (13.2131 µmol g−1 DW and 0.28 µmol g−1 DW, respectively). Mean anthocyanin content of the population represents a strong increase compared to the values found in both parents. As other assays previously described, anthocyanins presented transgressive distributions for both organs (Fig. 1). In the case of carotenoid content, differences between both organs were highly significant (F = 80.44, P = 0.012). Correlation coefficients among methods measuring AA, phenolic and pigment contents in the BolTBDH population were made. Pairwise correlations between AA measured with three ET assays (FRAP, DPPH and ABTS) were positive and highly significant (P≤0.01) for both leaves and flower buds in the correlation analysis carried out with all lines of the mapping population. However, correlation coefficients were moderately low (Table 2). The highest correlations occurred between DPPH and FRAP assays for both organs. The correlation values were 0.486 in flower buds and 0.526 in leaves. On the other hand, correlation coefficients between the content of phenolic compounds and the three AA methods were positive and significant for both organs (p≤0.01). Significant correlations between the anthocyanin content with DPPH and ABTS were found in leaves. Correlation with DPPH was positive; however, correlation with ABTS was negative (r =  −0.339, p≤0.01) (Table 2). Anthocyanin content was significantly and negatively correlated to ABTS assay (Table 2). Carotenoid content showed significant correlation coefficients with the AA measured with ABTS assay (r = 0.140, p≤0.05) in leaves, and significant and positive correlation coefficients with FRAP assay in flower buds (r = 0.305, p≤0.01). Furthermore, correlation between carotenoids and ABTS assay was negative and highly significant in flower buds (r =  −0.165, p≤0.01) (Table 2).

**Table 2:** Correlation coefficients for leaves (above the diagonal) and flower buds (below the diagonal) between the three antioxidant assay methods and the content of three metabolites with antioxidant activity (n = 280).

Leaves/Flower buds	ABTS	FRAP	DPPH	PHENOLICS	ANTHOCYANS	CAROTENOIDS
**ABTS**	–	0.197**	0.267**	0.434**	−0.339**	0.140*
**FRAP**	0.189**	–	0.526**	0.151*	0.103	0.100
**DPPH**	0.389**	0.486**	–	0.250**	0.164**	0.051
**PHENOLICS**	0.633**	0.221**	0.227**	–	−0.110	0.086
**ANTHOCYANINS**	−0.130*	−0.027	−0.076	−0.100	–	−0.081
**CAROTENOIDS**	−0.165**	0.305**	0.005	−0.013	0.176**	–

* Significant at p≤0.05, and ** significant at p≤0.01. ABTS: 2, 2′-azino-bis (3-ethylbenzthiazoline-6-sulphonic acid); FRAP: ferric ion reducing antioxidant power assay; DPPH: 2,2-diphenyl-1-picrylhydrazyl.

### QTL mapping for methods measuring AA, phenolic and pigment contents in the BolTBDH population

**Figure 2:**
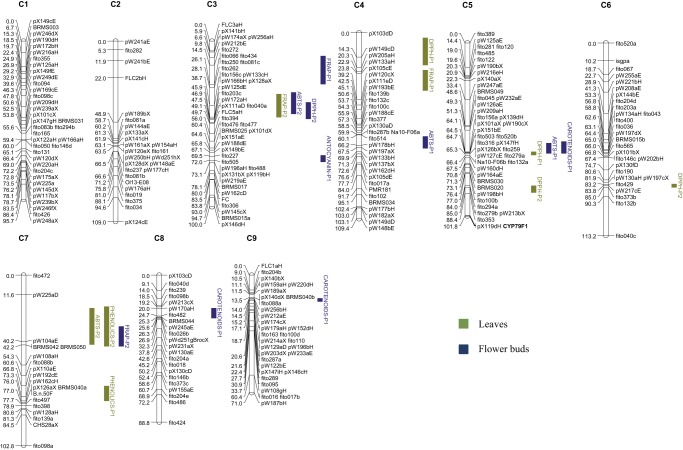
Framework map of DH population showing nineteen metabolic quantitative trait loci (QTL) for individual methods measuring AA. Linkage groups were labeled following the nomenclature of Iñiguez-Luy *et al.* [31]. Bars represent the LOD confidence interval of each QTL. QTLs are in different colors depending on the plant organ: leaves (green) and flower buds (blue). After the name of each QTL P1 indicates allele from, DH rapid cycling of Chinese kale (TO1000DH3) and P2 indicates allele from DH broccoli line ‘Early Big’.

A total of 19 QTLs were detected for all traits. The number of QTLs by linkage group ranged between one in C9 and five in C3 (Fig. 2). For methods measuring AA, seven significant QTLs were found in leaves. The value of R^2^ ranged between 9.8% for FRAP in C3 and 17.4% for DPPH in C4, respectively (Table 3). Three of these QTLs had a frequency of cross-validation higher than 50%. In flower buds, six significant QTLs were found. R^2^ value varied between 9.8% for ABTS in C6 and 12.1% for FRAP content in C3, but only two of the QTLs had a frequency of cross-validation higher than 50%.

**Table 3:** List of quantitative trait loci (QTL) for antioxidant activity and the content of metabolites with antioxidant activity in two plant organs under study, leaves and flower buds.

	Plant organ	Trait	Linkage group	Peak Position range (cM)	Left marker	Right marker	Lod theshold	LOD score	Frequency	Add	R^2^%	adj R^2^%
**1**	**Leaves**	**ABTS**	7	31 (20–42)	pW225aD	pW104aE	2.89	4.53	829	1.6354	14.2	12.5
**2**	**Leaves**	**DPPH**	4	12 (3–19)	pX103dD	pW149cD	2.85	5.45	972	−1.794	17.4	27.2
**3**			5	65 (64–66)	fito316, pX147fH, pX126bX, fito259, pW127cE & fito279a	Na10-F06b & fito132a		4.5	764	−2.112	14.1	
**4**			5	85 (84–88)	fito294a			3.83	605	2.202	12.2	
**5**			6	84 (83–85)	pW217cE	fito279b & pW213bX		3.64	249	3.455	11.6	
**6**	**Leaves**	**PHENOLICS**	7	34 (19–43)	pW225aD	pW104aE	3.02	3.23	278	0.294	10.4	10.5
**7**			7	73 (67–76)	pX110aE	pW192cE		3.2	213	−0.268	10.3	
**8**	**Leaves**	**FRAP**	3	41 (32–46)	fito156c, pW133cH, pW166bH & pX128aX	pW125dE	2.86	3.05	299	2.784	9.8	11
**9**			4	25 (22–34)	pX105cE	pW120cX		4.29	794	−2.518	13.5	
**10**	**Flower buds**	**ABTS**	3	38 (31–44)	fito262	fito156c, pW133cH, pW166bH & pX128aX	2.86	2.98	260	1.329	9.8	6.6
**11**			4	64 (61–68)	fito514	pW178bH		3.49	501	−2.441	11.4	
**12**			5	64 (59–65)	pW209aH	fito156a, pX139dH, pX101aX & pW190cX		3.28	348	−1.141	10.7	
**13**	**Flower buds**	**DPPH**	3	45 (37–47)	fito156c, pW133cH, pW166bH & pX128aX	pW125dE	2.86	3.11	48	1.402	10.2	1.3
**14**	**Flower buds**	**FRAP**	3	12 (9–26)	pW212bE	fito272	2.83	3.38	462	−2.325	11	6.8
**15**			7	40 (31–43)	pW225aD	pW104aE		3.72	631	2.538	12.1	
**16**	**Flower buds**	**Anthocyanin**	3	72 (69–73)	fito227	pW196aH, fito488, pX131bX, pX119bH & pW219aE	3.12	3.26	361	−4.458	10.9	0.2
**17**	**Flower buds**	**Carotenoid**	5	64 (58–65)	pW209aH	fito156a, pX139dH, pX101aX & pW190cX	2.93	2.94	226	−0.044	9.9	21.2
**18**			8	22 (20–26)	pW170aH	fito482		3.27	308	−0.047	10.9	
**19**			9	15 (14–16)	pW212aE	pW174cX		3.8	583	−0.049	12.6	

Additive effect was calculated as (P_2_–P_1_)/2; R^2^% coefficient of determination of each QTL. Adj R^2^%: determination coefficient of each trait.

For the content of metabolites with AA, two significant QTLs for phenolic content were found in leaves. The value of R^2^ ranged between 10.3 and10.4% in C7 and all of them had a frequency of cross-validation higher than 50%. Meanwhile, four significant QTLs were found in flower buds. The value of R^2^ ranged between 9.9 and 12.6% for carotenoids in C5 and C9, respectively. Only one of these QTLs presents a frequency of cross-validation higher than 50%. One QTL for anthocyanin content was found on C3 in flower buds, from which a R^2^ value of 10.9% and three QTLs for carotenoid content were found on C5, C8 and on C9. R^2^ values varied between 9.9 and 12.6% (Table 3).

Based on the position of QTLs and taking into account their confidence interval, three genomic regions determined variability for different traits. The genomic region located on C3, in the interval from marker pW125dE to fito156c & pW133cH (AA-C3), determined variation for the three different methods measuring AA: FRAP in leaves and ABTS and DPPH in flower buds. A second genomic region on C7 from pW225aD to pW104aE (AA-C7) determined variation for the methods measuring AA (ABTS in leaves and FRAP in flower buds) and phenolic content in leaves. Alleles for increasing AA or phenolic content are given by P2 in both genomic regions on C3 and C7. A third genomic region on C5 (AA-C5), from pW209aH to Na10-F06b & fito132a, also determined variation for the methods measuring AA (DPPH in leaves and ABTS in flower buds) and carotenoid content in flower buds. In this case, alleles for increasing AA and carotenoid content are given by P1.

Genes related to phenylpropanoid biosynthesis were located by means of *in silico* mapping in the confidence interval of several QTLs (Table 4). However no gene related to carotenoid biosynthesis could be located.

**Table 4:** List of phenylpropanoid biosynthesis candidate genes residing within the QTL confidence intervals according to organ and measurement method.

Plant organ	Trait	Markers in the confidence interval	Position in *Brassica oleracea* (cM) *	*Brassica oleracea* linkage group	Linkage group and position (bp) in *Arabidopsis thaliana* *	Genes related to phenylpropanoid biosynthesis located in the interval of *Arabidopsis thaliana*	Candidate genes
Leaves	FRAP	fito156c	38.72	3	1(3530200–3530221)		
		pW133cH	38.72	3	2 (15610858–15610982)		
		pX128aX	38.72	3	5 (6804683–6804766)		
		pW125dE	45.85	3	5(2219504–2219693)		
						AT5G48930	HCT
Flower buds	ABTS	fito250	36.12	3	5(15688217–26579698)		
						AT5G48930	HCT
Flower buds	DPPH	fito156c	38.72	3	1(3530200–3530221)		
		pW133cH	38.72	3	2 (15610858–15610982)		
		pX128aX	38.72	3	5 (6804683–6804766)		
		pW125dE	45.85	3	5(2219504–2219693)		
						AT5G48930	HCT
Flower buds	FRAP	pW212bE	9.81	3	3(6427399–6427450)		
		fito066	26.12	3	4(6017387–6017408)		
						AT4G00040	CHS and SS
Leaves	FRAP	pX105cE	23.78	4	2(16117201–18117509)		
						AT2G40890	CYP98A3
Leaves	DPPH	pW217cE	83.82	6	1(14257280–18257453)		
						AT4G30210	
Leaves	ABTS	fito472	0	7	4(18268924–18269031)		
		pW104aE	40.25	7	1(126837519–26837557)		
						AT1G51680	4CL
Leaves	phenolics	fito472	0	7	4(18268924–18269031)		
		pW104aE	40.25	7	1(126837519–26837557)		
						AT1G51680	4CL
Flower buds	FRAP	fito472	0	7	4(18268924–18269031)		
		pW104aE	40.25	7	1(126837519–26837557)	AT1G51680	4CL

* Candidate gene found by means of *in silico* mapping in the *Arabidopsis thaliana* TAIR database. CHS and SS: Chalcone and stilbene synthase family protein; 4-CL: 4-coumarate: Co-A ligase 1, 2 or 3; HCT: hydroxycinnamoyltransferase enzyme; C4H: cinnamate 4-hydroxylase.

## Discussion

### Quantitative variation for methods measuring AA and the content of metabolites with AA

Parents of the DH BolTBDH mapping population showed significant differences for the majority of the methods measuring AA and for the content of metabolites with AA in leaves and flower buds. BolTBDH population was found to be an ideal material in order to study QTLs for the traits under study in *Brassica* genus due to the differences between the two parents of this population. One parent (P2) is a broccoli ‘Early Big’ line, the *Brassica* crop with one of the highest AA [46], while the other parent (P1) is a DH rapid cycling line (TO1000DH3). Both parents are from different cultivars and as stated before, there is high variability for AA between different *Brassica* crops [16], [26], [34], [47].

The total AA of a sample can be measured by using several methodologies [15]–[17], [26]. The radical scavenging capacity of DH BolTBDH mapping population was measured by using three ET methods: ABTS FRAP and DPPH. The content of metabolites with AA like phenolics, anthocyanin and carotenoid was also measured. Some DH lines exhibited mean values of the traits falling between the values of the two parents, but others exhibited values which were extremely higher or lower than their parents. This phenomenon is referred to as transgressive segregation. Distributions of the methods measuring AA, phenolics and pigment content were, in most cases, transgressive. The action of complementary genes may be the primary cause of transgression, although epistasis may also contribute [48]. Further studies could help to explain the transgressive segregation of the traits measured in this study. These studies could use other populations or add more molecular markers to our population.

Total AA varied considerably according to the organ under study. Generally speaking, leaves present higher AA and content of metabolites with AA than flower buds, as it was expected by their photosynthetic complex. This result is in agreement with Soengas *et al.* [16] and Llorach *et al.* [49], who measured the AA of heads and leaves of cauliflower, with the highest values found in leaves. Guo *et al.* [50] found similar values in both organs in broccoli and Soengas *et al.* [16] found that broccoli flower buds have higher AA than leaves. In broccoli and cauliflower, the organs which are consumed are the heads (flower buds) and the leaves surrounding the heads are treated as by-products. Our results show that leaves have more AA and content of metabolites with AA than heads. Therefore, consumption of broccoli by-products, which is one of the parents of the mapping population, could be an interesting option to include in the human diet.

Due to the characteristics of the methods analyzed, AA measured with FRAP and DPPH assays present lower values compared to that of ABTS assay. It is coincident with the results found by Gouveia *et al.* [51] in other species like *Andryala glandulosa*.

### Correlation coefficients among methods measuring AA and the content of metabolites with AA

Significant correlation coefficients were found among the three methods measuring AA (FRAP, DPPH and ABTS) in the two organs under study, and ranged between 0.19 and 0.53. These correlations, although significant, were lower than others found in previous studies. Kusznierewicz *et al.* [17] found a correlation of 0.96 between ABTS and DPPH in white cabbage planted in different locations. Soengas *et al.* [16] found a correlation of 0.8 between DPPH and FRAP in extracts of different *Brassica* crops. Zhi *et *al. [18] found correlations ranging from 0.76 to 0.82 between the three cited methods analyzing different vegetables including broccoli. The material studied in our research is much closer genetically than the material studied in previously cited literature, since all the DH lines derive from a single cross. Clearly, correlations among ET methods depend on the material under study and based on our results, we recommend using more than one ET method in order to estimate the AA of a sample as suggested by Kurniereick *et al.* [17] and Gawlik-Dziki [52].

Significant correlations among the three methods measuring AA and the content of metabolites with AA were found in leaves and flower buds. Phenolic content was positively correlated with all the methods measuring AA. The correlation coefficient with ABTS showed the highest value in both organs. Several authors have found significant and high correlations (ranging from 0.7 to 1) between the AA measured with ABTS, DPPH and FRAP assays and phenolic content measured with the Folin–Ciocalteu method in other *Brassica* crops (cabbages, broccoli and Brussels sprouts) [15], [18], [26], [53], [54]. These results confirm the hypothesis that phenolic compounds mainly account for the AA of *Brassica* extracts. In the review made by Podsedek *et al.* [26], it is pointed out that phenolic compounds have higher AA in *in vitro* experiments than vitamins and carotenoids.

Furthermore, positive and significant correlations between carotenoid content and methods measuring AA were found in flower buds (FRAP) and in leaves (ABTS) in this study. These correlations are smaller than those of phenolic compounds with AA. Our results confirm that carotenoids are metabolites which contribute to the AA of Brassica extracts. Krinsky *et al.* [55] described that phenolic and carotenoid content is positively correlated with AA. In the case of anthocyanins, our experiments do not show a clear relationship between their content and methods measuring AA.

### QTL mapping for methods measuring AA and the content of metabolites with AA

Methods measuring AA on food extracts are extensively used by the scientific community in order to detect potential benefits for human health. Genetic variation for these traits is interesting from the breeder's points of view, since it could allow increasing the AA of *Brassica* foods by selection. As far as we know, no report of QTLs or genetic basis for methods measuring AA has been done before in any *Brassica* crop. This is also one of the first assays, which studies the genetic base of ET methods measuring AA in any crop. Only three recent pieces of research in rice [28], raspberry [29] and in lettuce [30] studied QTLs for total water AA, total phenolic content, anthocyanin and carotenoid content. Knowledge derived from this study can be utilized in order to search for genes underlying these traits.

Ten out of 19 QTLs determine AA or the content of metabolites with AA in only one of the two organs, thus indicating that the regulation of genes underlying several QTLs is organ-dependent. Seven QTLs determined variation for only one method measuring AA, thus indicating that the genetic basis regulation is partially dependent on the method. Genomic regions AA-C3, AA-C5 and AA-C7 determined variation for more than one ET method measuring AA. These genomic regions could be responsible for the significant correlations found between ET methods in this study.

The genomic region AA-C7 determines variation for methods measuring AA and phenolic compounds and the genomic region AA-C5 determines variation for methods measuring AA and carotenoid content. These finding supports the hypothesis that AA of the mapping population is related to phenolic compounds but also to carotenoid content. No QTLs related to methods measuring AA and anthocyanin content were found. Therefore, anthocyanins would not play a significant role in maintaining the AA of extracts in this population. The content of other compounds different from those under study could be responsible for the remaining QTLs, which control variation for methods measuring AA. The core reactions of phenylpropanoid metabolism involve several steps catalyzed by three key enzymes: phenylalanine ammonia-lyase (PAL), cinnamate 4-hydroxylase (C4H) and 4-Coumarate: CoA ligase (4CL) [56]. In *A. thaliana* there are 4CL different genes. This enzyme has a pivotal role in the biosynthesis of a plant's secondary compounds at the divergence point from general phenylpropanoid metabolism to several major branch pathways [57], [58]. After *in silico* mapping analysis, 4CL-1 gene was located in the genomic region AA-C7 which controls AA measured as ABTS and FRAP and phenolic content. The hydroxycinnamoyltransferase enzyme (HCT) appears to be potentially implicated in the pathway both upstream and downstream of the 3-hydroxylation step and it is another key enzyme in phenylpropanoid biosynthesis. HCT enzyme catalyzes reactions both immediately preceding and following the insertion of the 3-hydroxyl group into the monolignol pathway [59]–[61] realised by the CYP98A3 (C3′H). HCT gene from *A. thaliana* was located by means of *in silico* mapping in the genomic region AA-C3, which controls AA measured with the three ET methods. C3′H gene was located in the interval of pX105cE to pW120cX on chromosome 4 where a QTL for AA measured with FRAP method was found. More candidate genes related to phenylpropanoid biosynthesis, along all the linkage group, were identified as it is the case of the chalcone and stilbene (CHS and SS) family protein which catalyzed the initial steps for flavonoid biosynthesis, route related with the phenylpropanoid biosynthesis [62]. More work is necessary in order to validate and confirm candidate genes for the QTLs found in this study.

### Conclusions

No reports on the genetic basis of AA, and the content of metabolites with AA like phenolic, anthocyanin and carotenoid content have been documented before in *Brassica* crops. Results among methods measuring AA suggest that it is necessary to use more than one ET method in order to estimate AA, due to the fact that these methods present low significant correlations between them. Phenolic compounds and carotenoids are responsible for the AA of *Brassica* extracts.

Three genomic regions determined variation for more than one ET method measuring AA. QTL analysis confirms that AA of the mapping population is related to phenolic compounds but also to carotenoid content. It should be pointed out that the experiments have been carried on in one environment and under controlled conditions of temperature and light. Once the existence of QTLs for the traits under study has been proved, new experiments are going to be carried on in different environments to test the stability of the QTLs and the influence of environmental conditions. Several candidate genes related to phenylpropanoid biosynthesis are proposed for the QTLs found. These QTLs and the possible candidate genes identified through syntenic analysis with *A. thaliana* are the first step to understand the genetic basis of AA in the *Brassica* genus.
